# Low-Frequency Oscillations and Control of the Motor Output

**DOI:** 10.3389/fphys.2017.00078

**Published:** 2017-02-14

**Authors:** Neha Lodha, Evangelos A. Christou

**Affiliations:** ^1^Department of Health and Exercise Science, Colorado State UniversityFort Collins, CO, USA; ^2^Department of Applied Physiology and Kinesiology, University of FloridaGainesville, FL, USA

**Keywords:** force variability, force precision, voluntary drive, motor control, corticomuscular coherence

## Abstract

A less precise force output impairs our ability to perform movements, learn new motor tasks, and use tools. Here we show that low-frequency oscillations in force are detrimental to force precision. We summarize the recent evidence that low-frequency oscillations in force output represent oscillations of the spinal motor neuron pool from the voluntary drive, and can be modulated by shifting power to higher frequencies. Further, force oscillations below 0.5 Hz impair force precision with increased voluntary drive, aging, and neurological disease. We argue that the low-frequency oscillations are (1) embedded in the descending drive as shown by the activation of multiple spinal motor neurons, (2) are altered with force intensity and brain pathology, and (3) can be modulated by visual feedback and motor training to enhance force precision. Thus, low-frequency oscillations in force provide insight into how the human brain regulates force precision.

## Introduction

The force output always fluctuates around a mean value. This variability is detrimental to our ability to perform movements with accuracy, learn new motor skills, and use tools. *Why then does the central nervous system (CNS) allow the force output to fluctuate?* The prevalent answer is that force fluctuations represent “noise” derived from various parts of the (CNS) (Schmidt et al., 1979; Harris and Wolpert, 1998; Faisal et al., 2008). Nonetheless, emerging evidence in motor neuroscience suggest that force fluctuations are not random but rather strongly rhythmical (<1 Hz) (Slifkin and Newell, 1999, 2000; Slifkin et al., 2000; Vaillancourt and Newell, 2003; Sosnoff et al., 2009; Baweja et al., 2010; Fox et al., 2013; Lodha et al., 2013; Moon et al., 2014; Kang and Cauraugh, 2015). Given that oscillations of neuronal activity are fundamental to the communication between different parts of the CNS (Engel and Fries, 2010), force oscillations may represent one way the brain communicates with the spinal motor neurons. Alternatively, they may represent physiological rhythms (such as breathing) that perturb force control. In this review paper, we highlight that force fluctuations represent low-frequency oscillations of the spinal motor neuron pool from the descending drive. We demonstrate that oscillations below 0.5 Hz are detrimental to force precision but the CNS can modulate these oscillations by shifting power to higher frequencies. The evidence is summarized in the sections below.

## Significance of low-frequency oscillations in force

The conventional view is that force fluctuations represent noise in the CNS (Schmidt et al., 1979; Harris and Wolpert, 1998; Faisal et al., 2008). This view originated about 35 years ago (Schmidt et al., 1979) and was based on the tenets of information theory (Shannon, 1948). Classically, noise is defined as irregular fluctuations that perturb the transmitted signal (Shannon, 1948). It was hypothesized that Gaussian noise interfered with the neural signal of the voluntary motor command (Schmidt et al., 1979). The view of Gaussian noise has been challenged (Slifkin and Newell, 2000; Stergiou and Decker, 2011) because force variability (noise) is not proportional to the level of force (signal) (Slifkin and Newell, 1999, 2000; Christou et al., 2002) and because there is rhythmicity in the force output as identified by power spectrum analysis (Slifkin and Newell, 1999, 2000; Vaillancourt and Newell, 2003; Baweja et al., 2010; Fox et al., 2013; Lodha et al., 2013; Moon et al., 2014). Initial studies demonstrated that most of the power comes from low-frequencies <2 Hz (Slifkin and Newell, 1999, 2000; Vaillancourt and Newell, 2003; Sosnoff et al., 2009). However, these studies were limited by the 1 Hz resolution in their power spectral analysis in identifying whether other peaks exist within these bands.

**Figure 1:**
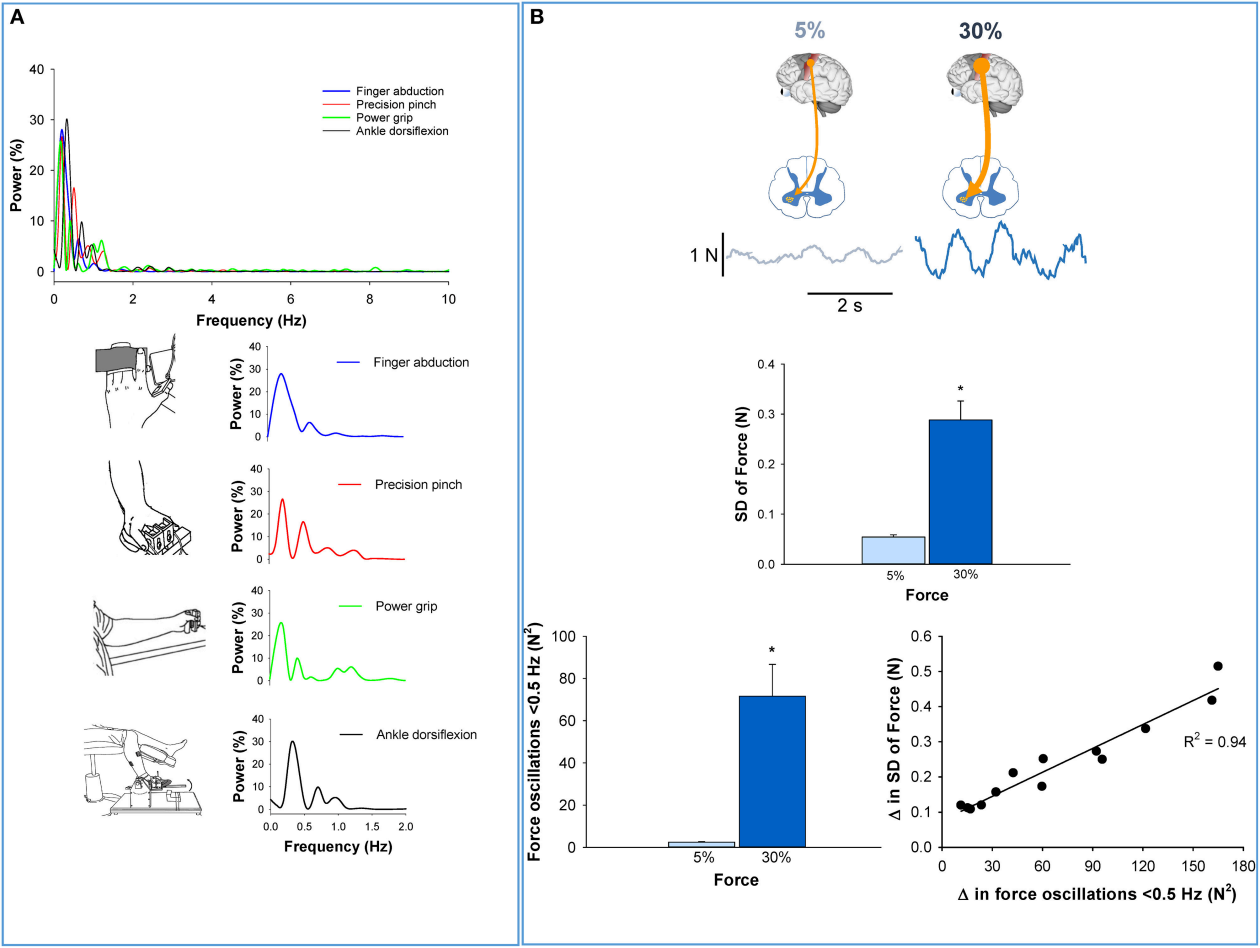
**Low-frequency oscillations in the force output. (A)** The power spectrum of force during various constant contractions (Top). Regardless of the effector performing the task, the majority of the power occurs below 1 Hz. Specifically, over 80% of the power for finger abduction, pinch grip, power grip, and ankle dorsiflexion occurs below 1 Hz. In addition, the peak oscillation is observed below 0.5 Hz. *These findings suggest that low-frequency oscillations in force are independent of the type (upper or lower limb) or number (single or multi-digit) of effectors that are necessary to perform the task*. **(B)** Top row shows that the voluntary drive from the higher centers to the spinal motor neurons increases when young adults exert a higher amount of force from 5% (light blue) to 30% (dark blue) MVC (top row) (Neto et al., 2010). The middle row shows that force variability increased with stronger voluntary drive (from 5 to 30% MVC). In the bottom row, we show that oscillations in force below 0.5 Hz also increased with higher voluntary drive (30% MVC). The increase in force variability with voluntary drive was strongly related (*R*^2^ = 0.94, *p* < 0.05; Moon et al., 2014) to increased oscillations in force below 0.5 Hz (bottom row–right). *Thus, this figure shows that oscillations in force below 0.5 Hz increase with the strength of the voluntary drive and are detrimental to force precision*.

Our recent studies (Fox et al., 2013; Lodha et al., 2013; Moon et al., 2014), which incorporated a resolution of 0.1 Hz, clearly demonstrate that most of the fluctuations are a consequence of oscillations below 1 Hz. Specifically, oscillations below 0.5 Hz explained ~80% of force variability. In Figure 1A, we provide examples that oscillations below 1 Hz dominate the force output during single muscle contractions (abduction of the index finger; Moon et al., 2014) and multiple muscle contractions (such as pinch grip and power grip; Lodha et al., 2013). In addition, low frequency oscillations are evident in both the upper limb (e.g., abduction of the index finger, pinch grip, and power grip) and lower limb contractions (e.g., ankle dorsiflexion; Kwon et al., 2011). These results suggest that the low-frequency oscillations are present in the force output regardless of the number or the type of effectors used. The force output, therefore, is not irregular (Gaussian noise) but highly rhythmical.

This rhythmicity may represent a strategy by the CNS to regulate the force output or a physiological oscillation that perturbs the motor command (see section below). Regardless, the functional significance of low-frequency oscillations is the precision of the force output. Specifically, force oscillations below 0.5 Hz are positively related to force variability (decreased force precision; Fox et al., 2013; Lodha et al., 2013; Moon et al., 2014). Supporting evidence comes from experiments that examine the effect of the strength of voluntary drive on force precision, compare force precision in healthy young and older adults, and examine the effect of neurological disorders on force precision. The evidence that low-frequency oscillations in force are related to force precision is presented below.

### Voluntary drive

A well-accepted phenomenon in motor neuroscience is that force precision decreases with increased voluntary drive (Schmidt et al., 1979; Slifkin and Newell, 2000; Christou et al., 2002). Recently, we examined whether this decrease in force precision with increased voluntary drive was related to changes in the oscillations in force below 0.5 Hz (Moon et al., 2014). We found that when healthy young adults increased the voluntary drive from 5 to 30% of their maximum voluntary contraction (MVC), they exhibited less force precision (Figure 1B, middle) and greater oscillations in force below 0.5 Hz (Figure 1B, bottom left). Most importantly, the decrease in force precision with voluntary drive was strongly related to increased oscillations in force below 0.5 Hz (Figure 1B, bottom right). These results suggest that force oscillations below 0.5 Hz are strongly related to the declines in force precision with voluntary drive.

### Aging

**Figure 2:**
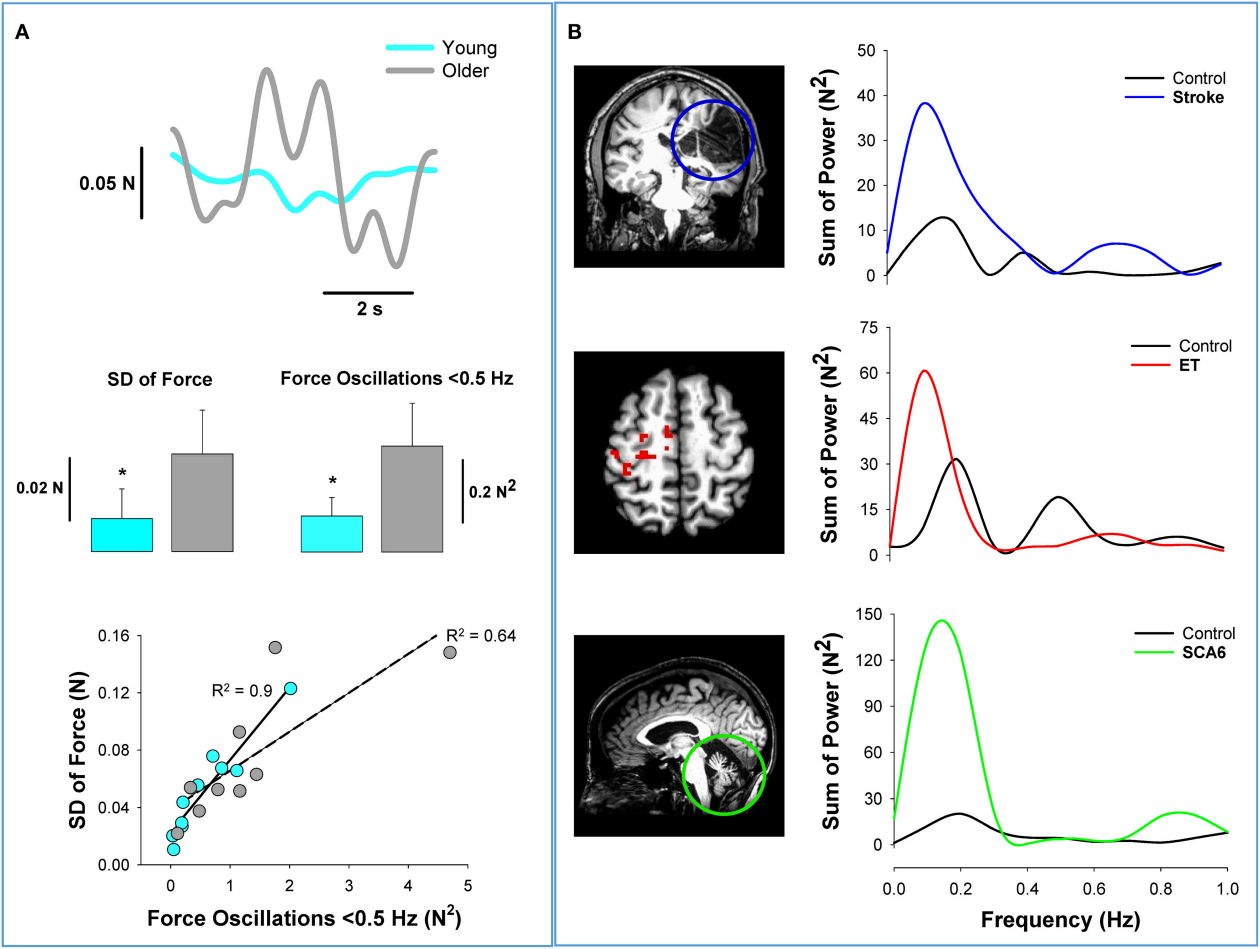
**Low-frequency oscillations in aging and neurological disorders**. Healthy aging and neurological disorders induce significant structural and functional changes in the brain. This figure shows how low-frequency oscillations increase with aging and brain pathology. **(A)** Force variability is greater in healthy older adults than young adults. The top row shows the variability in the force output from a young (light blue) and an older adult (gray) during a fine constant force contraction (2% maximum). In the middle row, we show that older adults exhibit greater variability of force (left) and greater oscillations in force below 0.5 Hz (right) (Fox et al., 2013). The bottom row shows that the oscillations in force contribute significantly to the force variability in both young (*R*^2^ = 0.9) and older adults (*R*^2^ = 0.64). **(B)** The argument that force oscillations below 0.5 Hz likely originate at the brain is supported from similar findings in patients with brain pathology. In the top row, we provide an example from stroke (Lodha et al., 2013). The coronal slice of the structural MRI scan shows a significant loss of cortical neurons (blue circle) in an individual post-stroke. The power spectrum of grip force (5% MVC) shows that the oscillations in force below 0.5 Hz are dominant in both the individual with stroke (blue) and healthy adult (black). Most importantly, the power from 0 to 0.5 Hz appear to quadruple following stroke. In the middle row, we provide an example from essential tremor (Neely et al., 2015). The axial slice of functional MRI scan shows significant cortical hyperactivity in individuals with essential tremor compared with healthy individuals. The power spectrum of pinch grip force shows that the oscillations in force below 0.5 Hz are dominant in both the individual with essential tremor (red) and the healthy adult (black). The power from 0 to 0.5 Hz appears to be double in the patient with essential tremor. In the bottom row, we provide an example from spinocerebellar ataxia type 6 (SCA6) (Casamento Moran et al., 2015). The sagittal slice of the structural MRI scan shows significant isolated cerebellar degeneration in SCA6. The power spectrum of ankle dorsiflexion force shows that the oscillations in force below 0.5 Hz are dominant in both the individual with SCA6 (green) and the healthy adult (black). The power from 0 to 0.5 Hz appears to be five times greater in the individual with SCA6. *Together, the data from healthy older adults and from the individuals with brain pathology, suggest that the origin and regulation of oscillations in force below 0.5 Hz occurs at the brain level*.

It is a well-documented finding that force precision declines with healthy aging (Enoka et al., 2003; Christou, 2011). Previous work from our lab examined whether the age-associated reduction in force precision is related to oscillations in force below 0.5 Hz (Fox et al., 2013). We found that older adults exhibited less force precision and greater oscillations in force below 0.5 Hz relative to young adults (Figure 2A). In Figure 2A, we show that older adults exhibit impaired force precision (top row and middle row–left) and greater power in force oscillations below 0.5 Hz than young adults (middle row–right). In both groups, impaired force precision was strongly related to increased oscillations in force below 0.5 Hz (bottom row). These results suggest that the force oscillations below 0.5 Hz contribute to reduced force precision in older adults.

### Neurological disorders

Low-frequency oscillations in force are evident in populations with neurological disorders that exhibit decreased force precision (Lodha et al., 2013; Kang and Cauraugh, 2015). Specifically, individuals with stroke (Lodha et al., 2013), essential tremor (ET) (Neely et al., 2015), and spinocerebellar ataxia (SCA6) (Maschke et al., 2004; Casamento Moran et al., 2015) exhibit increased low-frequency oscillations and decreased force precision relative to healthy controls. In Figure 2B, we demonstrate that these three neurological populations exhibit greater oscillations in force below 0.5 Hz. In the top row of Figure 2B, we compare the force output from an individual post-stroke to that of a matched healthy participant (Lodha et al., 2013). It is clear from the MRI that stroke induces a significant loss of cortical neurons. The power spectrum of the force output from a power grip demonstrates that the oscillations below 0.5 Hz are significantly greater in individual post-stroke. We have demonstrated previously in individuals post-stroke that low-frequency oscillations contribute to their impairments in force precision (Lodha et al., 2013). In the middle row of Figure 2B, we compare the force output from an individual with ET to a healthy participant (Neely et al., 2015). The functional MRI demonstrates the hyperactivity of the contralateral cortex for individuals with ET relative to healthy controls. The power spectrum of the force output during a pinch grip task performed by an individual with ET shows higher power below 0.5 Hz in force. Finally, in the bottom row of Figure 2B, we compare the force output from an individual with type 6 spinocerebellar ataxia (SCA6) to a healthy participant (Casamento Moran et al., 2015). It is evident from the MRI that the individual with SCA6 shows significant isolated degeneration of the cerebellum. The power spectrum of the force output during ankle dorsiflexion suggests that the SCA6 individual has higher power below 0.5 Hz compared with the healthy participant. These results advocate that disorders affecting brain regions amplify oscillations in force below 0.5 Hz, which contribute to reduced force precision.

In summary, oscillations in force below 0.5 Hz are present in the motor output during different muscle contractions and contribute to the decline in force precision with increased voluntary drive, aging, and neurological disease. Thus, low-frequency oscillations may potentially represent a fundamental way that our CNS controls the force output. Given that force oscillations are modulated with voluntary drive and influenced with brain pathology, the modulation of low-frequency oscillations in force likely originates in brain centers. In the section below we provide evidence that the low-frequency oscillations in force represent oscillations of the spinal motor neuron pool from the descending drive (supraspinal centers).

## Origins of low-frequency oscillations in force

Neuronal oscillations is a well-studied phenomenon (Engel et al., 2001; Engel and Fries, 2010). For example, the firing of cortical neurons, examined often with electroencephalography (EEG), is described with five frequency bands. The lowest band is delta and occurs from 0.5 to 3.5 Hz. Delta oscillations are associated with learning, motivation, reward system and deep sleep (Steriade et al., 1993; Knyazev, 2007). The theta (4–7 Hz) and alpha (8–12 Hz) bands are associated with working memory, short-term memory, and emotional arousal (Steriade et al., 1993). Finally, the beta (13–30 Hz) and gamma (>30 Hz) bands are primarily related to motor function (Engel and Fries, 2010). The beta band is important for the maintenance of a steady motor output (Engel and Fries, 2010), whereas the gamma band is related to sensorimotor integration and movement preparation and execution (Kristeva-Feige et al., 2002). Therefore, it appears that the brain primarily communicates with the spinal motor neuron pool through oscillations from 10 to 60 Hz.

The synchronized discharge of cortical and spinal neurons, is observed with the coherence between EEG or MEG and contralateral EMG signals (corticomuscular coherence) (Mima and Hallett, 1999a,b). Corticomuscular coherence, as it relates to motor tasks, is primarily seen from 10 to 60 Hz. When the task output requires steadiness, corticomuscular coherence is strong in the beta band (10–30 Hz). The beta band corticomuscular coherence has been demonstrated in monkeys (Baker et al., 1999) and humans (Conway et al., 1995; Halliday et al., 1998; Kilner et al., 2000; Kristeva et al., 2007). In contrast, during dynamic contractions, the corticomuscular coherence shifts to higher frequencies from 30 to 60 Hz (gamma band; Kristeva-Feige et al., 2002). Although corticomuscular coherence occurs from 10 to 60 Hz, the force output fluctuates below 1 Hz. *Thus, an important but unresolved question is “how higher frequency oscillations seen in corticomuscular coherence transform into low-frequency oscillations in the force output?”*

**Figure 3:**
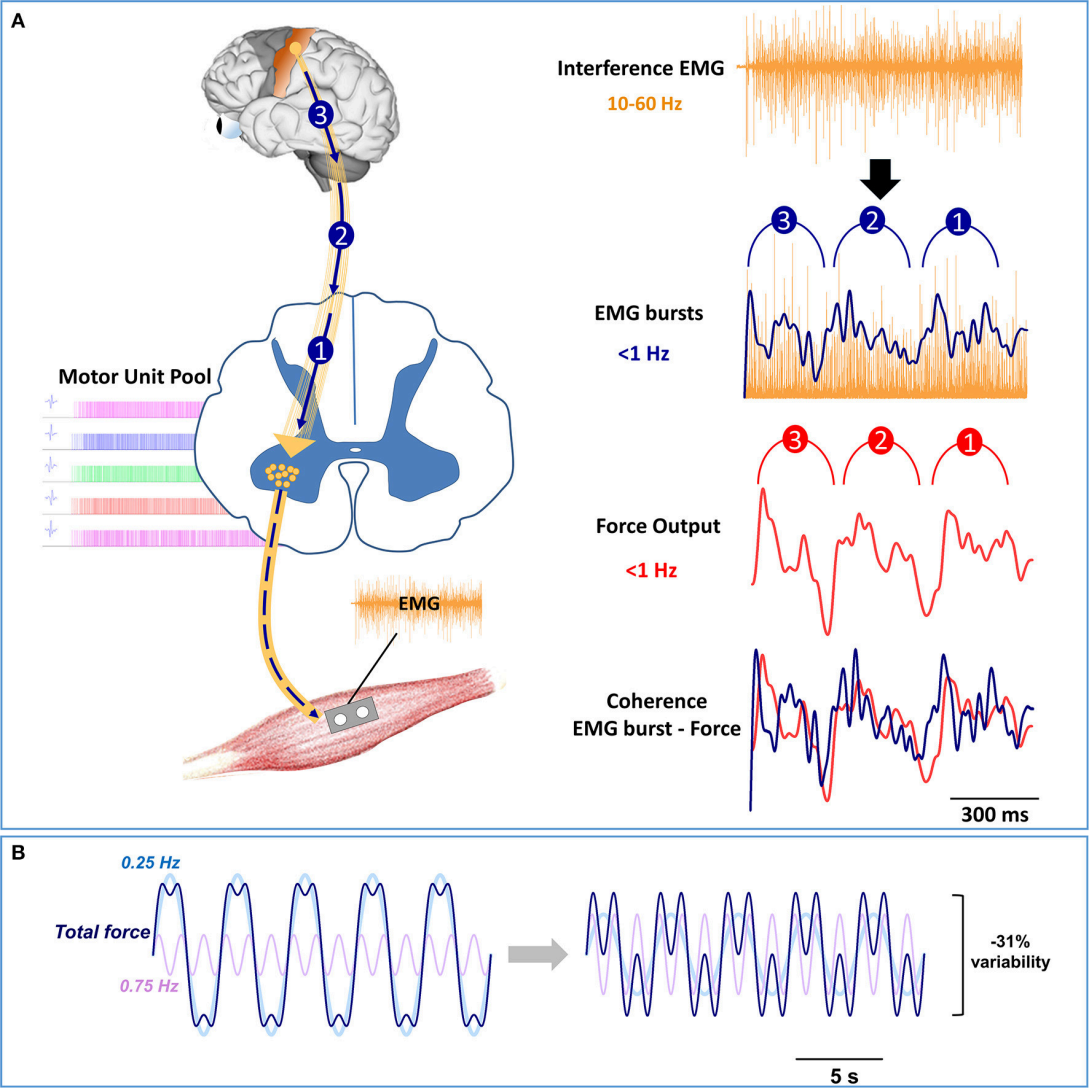
**Model pathway and modulation of low-frequency oscillations. (A)** On the left, the oscillations in the voluntary drive from 10 to 60 Hz are represented with gold lines from the motor cortex to the spinal motor neuron pool, whereas the low-frequency oscillations are represented as 3 blue arrows. The oscillations in the voluntary drive will activate multiple motor units (motor unit pool) and consequently the whole muscle. On the right, we show how the voluntary drive to the spinal motor neuron pool induces low-frequency oscillations in force. The modulation of whole muscle activity from 10 to 60 Hz is evident in the interference EMG signal (top row). However, to observe the modulation of whole muscle activity at frequencies below 1 Hz requires EMG processing (2nd row). Specifically, to identify the bursts in the activity of the motor units, the interference EMG must be rectified and low-pass filtered (<5 Hz). Recent experiments in our lab (Moon et al., 2014) suggest that EMG bursts occur at frequencies below 0.5 Hz, with the strongest oscillation at ~0.25 Hz. Most importantly, the low-frequency oscillations in EMG bursting are coherent with force oscillations (3rd row) and precede force oscillations with a constant time (~29 ms) (4th row). **(B)** Based on recent experimental findings in our lab (Fox et al., 2013; Lodha et al., 2013; Moon et al., 2014), we simulated the force output with oscillations below (0.25 Hz) and above 0.5 Hz (0.75 Hz). The total fluctuations in the force output (dark blue line) represent the sum of two force oscillations, 0.25 Hz (light blue line) and 0.75 Hz (light purple line). On the left, the total fluctuations in force are represented with the following equation: $f1\left(x\right)=4sin\frac{\pi }{2}+sin\frac{3\pi }{2}$ On the right, we decrease the total force fluctuations by modulating the amplitude of the force oscillations with the following equation: $f2\left(x\right)=2sin\frac{\pi }{2}+2sin\frac{3\pi }{2}$ This Figure shows that halving the power in the 0.25 Hz and doubling the power in the 0.75 Hz force oscillations, decreases force fluctuations by 31%.

One proposition is that the higher frequency oscillations to the spinal motor neurons are organized in low-frequency bursts of multiple motor units. The coherent low-frequency modulation of multiple motor units with force has been demonstrated previously (De Luca et al., 1982a,b; De Luca, 1985; Negro et al., 2009; Yoshitake and Shinohara, 2013). For example, Farina and colleagues [38], showed that the summed activity of 9 individual motor units across 400 ms was related to force oscillations. Recently, we demonstrated that oscillations in multiple motor units, quantified with whole muscle electromyography (EMG), manifest bursts below 0.5 Hz and are coherent with force oscillations (Moon et al., 2014; see Figure 3A). Interestingly, such low-frequency bursts in muscle activity were positively related to higher-frequency oscillations in whole muscle activity (35–60 Hz). Figure 3 graphically shows a model pathway for the low-frequency oscillations. The oscillations in the voluntary drive (10–60 Hz) are transmitted form the motor cortex to the spinal motor neuron pool that activate the whole muscle at frequencies below 1 Hz. Thus, low-frequency oscillations at the neural level may be embedded in the timing of the bursts of multiple motor units.

This finding raises the following important question: *Why does the descending drive contain low-frequency oscillations?* One possibility is that it represents a strategy by the CNS to efficiently regulate the precision of the force output. This strategy solves the abundant degrees of freedom problem first introduced in motor neuroscience by Bernstein (1967). In this instance, the problem would be that the CNS must control hundreds of spinal motor neurons individually to regulate the force output, which could be very demanding and inefficient (for an extensive discussion/review on this issue see Latash, 2012). Instead of controlling individual spinal motor neurons, the CNS groups the activity of the spinal motor neurons into low-frequency units. Thus, low-frequency oscillations in the descending drive may reflect a way to regulate force precision.

Another possibility is that the low-frequency oscillations in the spinal motor neuron pool reflect physiological rhythms from cortical activity and sympathetic nervous system activity. These oscillations can be viewed as “noise” to the activation of the spinal motor neuron pool because they perturb the motor command. Recent evidence suggests that cortical neurons in the pre-motor cortex and motor cortex oscillate at a very low frequency and perturb the performance of goal-directed movements (Chaisanguanthum et al., 2014). In addition, physiological rhythms are evident in the sympathetic motor system, which regulates breathing, heart rate, and blood pressure (Croix et al., 1999). In humans, muscle sympathetic nervous system activity (MSNA), is dominated by oscillations that are related to breathing (~0.25 Hz) and heart rate (~1 Hz) (Croix et al., 1999). Consequently, low-frequency oscillations related to breathing could potentially disturb force control. This has been demonstrated in orofacial muscles and speech (Moore et al., 2014) and more recently during dexterous finger control (Baweja et al., 2011). Further, the potential influence of MSNA on force control is demonstrated with the increase of MSNA with physiological stress (Victor et al., 1987; Callister et al., 1992). We have shown that increased physiological stress amplifies low-frequency oscillations in force (~1 Hz) and impairs force precision (Christou et al., 2004). Regardless of whether the low-frequency oscillations in the spinal motor output reflect a strategy of the CNS or perturbation from physiological rhythms, the evidence points to the central origin of low-frequency oscillations.

We propose that oscillations in force below 0.5 Hz are related to the voluntary drive from higher centers (Figure 3A). This proposition is derived primarily from the following three sets of findings: First, low-frequency oscillations in force are affected by brain pathology (see Figures 2B). Second, low-frequency oscillations in force are modulated with force level (Moon et al., 2014). An increase in force level requires a stronger voluntary drive from higher centers (Neto et al., 2010) without changes to the peripheral nervous system (e.g., muscles and reflexes). Third, low-frequency oscillations in force are modulated with processing of visual information (Baweja et al., 2010; Fox et al., 2013). We found that increasing visual information reduced the force oscillations below 0.5 Hz and increased force oscillations above 0.5 Hz (Fox et al., 2013). Together, the modulation of low-frequency oscillations with brain pathology, stronger voluntary drive, and increased visual information support the idea that low-frequency oscillations in force output have a central origin.

## Modulation of low-frequency oscillations in force

*How can the CNS constrain the low-frequency oscillations in force and enhance force precision?* Recent findings suggest that low-frequency oscillations induce the largest fluctuations in the force output and impair force precision (Fox et al., 2013; Lodha et al., 2013; Moon et al., 2014). Thus, one way for the CNS to enhance force precision is to shift the power from low frequencies to higher frequencies in force (Fox et al., 2013; Lodha et al., 2013). Based on recently published data (Moon et al., 2014), this concept is demonstrated theoretically in Figure 3B. We simulated a fluctuating force output (dark blue line) comprised of two oscillatory signals, 0.25 Hz (light blue line), and 0.75 Hz (light purple line). When the power in the 0.25 Hz signal decreases to 50% and the power in the 0.75 Hz doubles, the oscillations in the total force decrease by ~30%. In the section below we provide experimental evidence that low-frequency oscillations in force can be modulated by increasing the frequency of oscillation with visual feedback and motor training.

**Figure 4:**
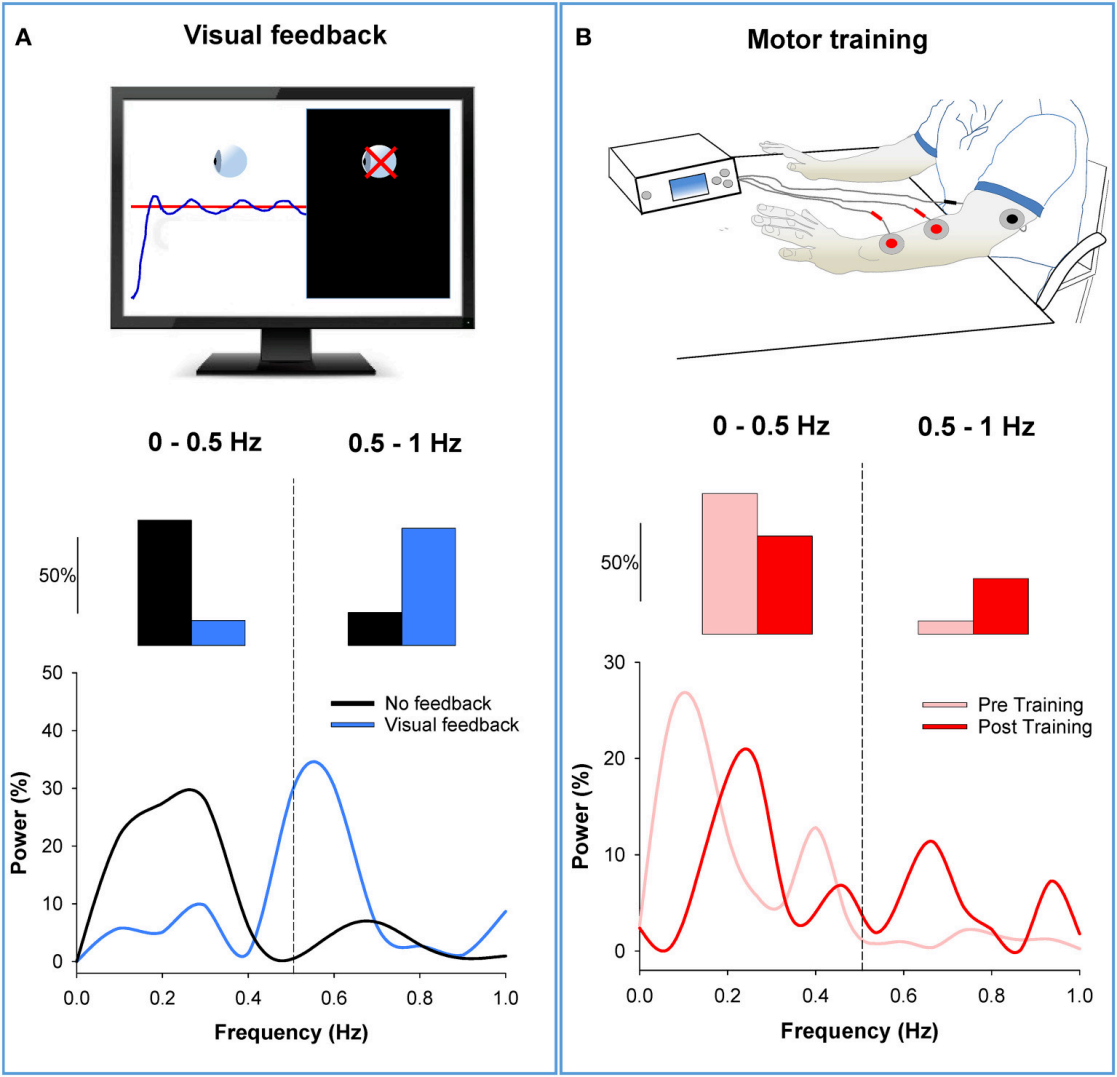
**Modulation of low-frequency oscillations in the force output. (A)** In the top row, we show an isometric force contraction performed with and without visual feedback (black screen on the right; Fox et al., 2013). In the middle row, we show that power from 0 to 0.5 Hz decreased with visual feedback (blue bar) compared with no visual feedback (black bar). In contrast, power from 0.5 to1 Hz increased with visual feedback. The bottom row shows the power spectrum of the force output. It shows that the power shifts from low to high frequencies with visual feedback (blue line) relative to no visual feedback (black line). **(B)** In the top row, we show the bilateral motor training (Kang and Cauraugh, 2014). Individuals with chronic stroke practiced voluntary movements with both arms while the paretic arm received EMG-triggered neuromuscular stimulation for 6 weeks. In the middle row, we show that power from 0 to 0.5 Hz decreased post motor training (red) compared with pre training (pink). In contrast, power from 0.5 to 1 Hz increased post training compared with pre training. The bottom row shows the power spectrum of the force output. It shows that the power shifts from low to high frequencies post training (red line) compared with pre training (pink line). *Thus, this figure shows that visual feedback and motor training can reduce oscillations in force below 0.5 Hz by shifting the power to higher frequencies*.

There is evidence that varying the amount of visual feedback can influence low-frequency oscillations in force output (Baweja et al., 2010; Fox et al., 2013). Most importantly, we found that visual feedback differentially modulates power in force oscillations from 0 to 0.5 Hz and 0.5 to 1 Hz (Fox et al., 2013). Force precision improved with magnification of visual feedback when participants decreased power below 0.5 Hz and increased power above 0.5 Hz (see Figure 4). The results from the visual feedback experiment suggest two important things: (1) Not all oscillations below 1 Hz are detrimental to force precision, and thus should not be grouped into one band; (2) visual feedback can be an important tool to reduce force oscillations below 0.5 Hz and consequently improve force precision.

Support for the idea that a shift of power at low-frequencies improves force precision comes from a recent training study in individuals with stroke (Kang and Cauraugh, 2014). Specifically, 15 individuals post-stroke underwent bilateral motor training for 6 weeks. This type of training involved practice of voluntary movements with both arms while the paretic arm received EMG-triggered neuromuscular stimulation. The force precision and function (grasp and release) of the paretic hand in individuals post-stroke improved significantly following the bilateral motor training. The training-induced improvement in force precision was related to decreased power from 0 to 0.5 Hz and increased power from 0.5 to 1 Hz in the force output. In Figure 4B, we provide an example from one participant with stroke before and after the bilateral motor training. Following training, power in the force output decreased from 0 to 0.5 Hz and increased from 0.5 to 1 Hz. These results validate that a shift in power from low (0–0.5 Hz) to high (0.5–1 Hz) frequency oscillations mediate improvements in force precision.

Collectively, these studies support the idea that visual feedback and motor training may modulate low-frequency oscillations in force and enhance force precision. Both types of interventions appear to reduce oscillations below 0.5 Hz by shifting the power to 0.5–1 Hz band. Therefore, training protocols that target improvements in force precision must constrain oscillations in force below 0.5 Hz.

## Concluding remarks and future directions

Commonly, force fluctuations are regarded as noise derived from various parts of the CNS and harmful to force precision. Here, we summarize the current evidence in literature showing that the primary source of force fluctuations is strongly rhythmical and occurs at a frequency below 0.5 Hz. Therefore, force fluctuations are not noise in the traditional sense (random variations around the signal) but rather rhythmical events in the force output. Based on the evidence presented, these low-frequency oscillations are embedded in the descending drive and induce low-frequency bursts in multiple motor unit activity. What remains unclear is whether the low-frequency oscillations reflect a CNS strategy to regulate force precision or simply reflect a rhythmicity in the CNS that perturbs the motor command.

If these low-frequency oscillations indeed reflect a strategy, then the CNS must be able to benefit from their modulation. Theoretically, the CNS becomes more efficient by grouping the activity of spinal motor neurons into bursts of low-frequency oscillations so that it does not have to control each neuron independently. In application, the regulation of these low-frequency oscillations appears to improve force precision. We showed that force precision improves when the CNS reduces the oscillations below 0.5 Hz and shifts power to higher frequencies (0.5–1 Hz).

Future studies should distinguish whether low-frequency oscillations reflect a CNS strategy or a rhythmical perturbation to the voluntary motor command. If it is a perturbation, then reduction of power in low-frequency oscillations will result in better motor performance regardless of the task requirement. If it's a strategy, then low-frequency oscillations will be modulated differently based on task requirements (e.g., precision vs. speed). For example, to accomplish a precise contraction the CNS may increase the beta drive (10–35 Hz) and reduce the low-frequency oscillations. In contrast, to accomplish a fast contraction that does not require precision, the CNS may increase the gamma drive (35–60 Hz) and amplify the low-frequency oscillations [8]. Finally, to detect low-frequency oscillations below 1 Hz, an important methodological consideration for future studies is to examine power spectrum of the force output with high resolution (e.g., 0.1 Hz). Such studies will have tremendous implications to our understanding of the motor system and to the design of rehabilitation protocols for individuals with impaired motor control, such as older adults and patients with neurological disorders.

## Author contributions

NL and EC provided contributions to the conception or design of the work; the acquisition, analysis, or interpretation of data for the work; and Drafting the work for important intellectual content; and Final approval of the version submitted; and Agree to be accountable for all aspects of the work in ensuring that questions related to the accuracy or integrity of any part of the work are appropriately investigated and resolved.

### Conflict of interest statement

The authors declare that the research was conducted in the absence of any commercial or financial relationships that could be construed as a potential conflict of interest.
